# HBsAg loss in chronic hepatitis B patients with an HBsAg decline plateau can be enhanced by intermittent PEG-IFN treatment

**DOI:** 10.3389/fcimb.2026.1778016

**Published:** 2026-05-21

**Authors:** Weihua Cao, Si Xie, Hongxiao Hao, Wen Deng, Lu Zhang, Yao Lu, Ruyu Liu, Shiyu Wang, Xinxin Li, Ziyu Zhang, Xin Wei, Zixuan Gao, Linmei Yao, Shuojie Wang, Yuanjiao Gao, Yujie Wang, Mengjiao Xu, Leiping Hu, Yao Xie, Minghui Li

**Affiliations:** 1 Department of Hepatology Division 2, Beijing Ditan Hospital, Capital Medical University, Beijing, China; 2 HBV Infection, Clinical Cure and Immunology Joint Laboratory for Clinical Medicine, Capital Medical University, Beijing, China; 3 Division of Hepatology, Hepato-Pancreato-Biliary Center, Beijing Tsinghua Changgung Hospital, School of Clinical Medicine, Tsinghua University, Beijing, China; 4 Department of Hepatology Division 2, Peking University Ditan Teaching Hospital, Beijing, China

**Keywords:** antiviral therapy, chronic hepatitis B, HBsAg loss, intermittent therapy, pegylated interferon

## Abstract

**Background:**

During interferon (IFN) treatment patients with chronic hepatitis B (CHB), HBsAg levels often declines to a plateau. This study investigated the HBsAg loss rate in patients receiving continuous IFN treatment after reaching a plateau compared with patients who suspended IFN treatment for 3–6 months.

**Methods:**

Patients were enrolled from an observational cohort of CHB patients receiving PEG-IFN-α-based therapy. All patients reached a plateau in qHBsAg decline after 24 weeks of PEG-IFN-α treatment. Patients in the continuous-treatment group received continuous PEG-IFN therapy, while patients in the intermittent-treatment group discontinued initial PEG-IFN therapy for 3–6 months before resuming treatment.

**Results:**

In this retrospective study, 403 CHB patients with a plateau in HBsAg decline after 24 weeks of pegylated IFN-α (PEG-IFN-α) treatment were included. After reaching the plateau, patients either continued PEG-IFN treatment or restarted treatment after an interval. The HBsAg loss rate in the intermittent-treatment group was significantly higher than that in the continuous-treatment group (p = 0.001); in the 278-case propensity score matching (PSM) population, HBsAg loss rates were 21.58% and 8.63%, respectively (p = 0.002). Multivariate Cox regression analysis showed that intermittent therapy (p = 0.017) and HBsAg level at the second plateau time point (p < 0.001) were independent factors of HBsAg loss. Multivariate Cox regression analysis of the PSM-matched population showed that baseline HBsAg level (p = 0.043), HBsAg level at the second plateau time point (p = 0.000), and intermittent therapy (p = 0.004) were independent factors of HBsAg loss.

**Conclusions:**

Among CHB patients receiving PEG-IFN-based treatment, intermittent treatment was more beneficial than continuous treatment in achieving HBsAg loss once HBsAg decline reached a plateau.

## 1 Introduction

Owing to the successful use of antiviral therapy with lamivudine (LAM) in patients with chronic hepatitis B (CHB), mortality of patients with hepatic failure induced by chronic hepatitis B virus (HBV) infection has decreased over time. However, mortality of patients with hepatocellular carcinoma (HCC) caused by HBV infection has gradually increased (Liu et al., 2019; Chinese Society of Hepatology, Chinese Society of Infectious diseases, 2022). The five-year cumulative incidence rate of HCC in CHB patients who achieve HBsAg loss is less than 1.0% (Yip et al., 2019). CHB patients who achieve HBV DNA negativity together with HBsAg and HBeAg seroconversion are considered to have the strongest immune control over HBV infection (van Campenhout and Janssen, 2015). Therefore, treatment strategies aimed at achieving HBsAg clearance are recommended in current guidelines (European Association for the Study of the Liver, 2017; Terrault et al., 2018; Chinese Society of Hepatology, Chinese Society of Infectious Diseases, 2022).

Serum HBsAg levels in the peripheral blood of CHB patients are associated with both the quantity and transcriptional activity of cccDNA in the liver (Chan et al., 2007; Brunetto, 2010). HBsAg levels may also reflect clearance of virus-infected hepatocytes (Moucari et al., 2009; van Campenhout and Janssen, 2015). In interferon (IFN)-treated CHB patients, the clearance half-life of virus-infected hepatocytes varies substantially (Sypsa et al., 2005), and more than 75% of patients who achieve HBsAg loss require treatment longer than 48 weeks (Li et al., 2021). Prolonged therapy is a vital measure to improve HBsAg loss rates in CHB patients (Li et al., 2017a; Pan et al., 2021). In clinical practice, patients with HBsAg loss often show an early and rapid decline in HBsAg levels. However, these patients do not necessarily achieve HBsAg loss after prolonged pegylated IFN (PEG-IFN) treatment. During IFN therapy, the decline in HBsAg levels usually reaches a plateau, after which prolonged treatment does not further decrease HBsAg levels [Li et al., 2021].

In this study, an HBsAg decline plateau was defined as a qHBsAg decline of less than 0.5 log IU/mL between two consecutive visits separated by a minimum interval of three months after PEG-IFN-α treatment. When patients reached the HBsAg decline plateau after PEG-IFN treatment, they had two PEG-IFN treatment options: extend the PEG-IFN treatment (continuous-treatment model) or suspend PEG-IFN treatment for an interval and then resume PEG-IFN retreatment (intermittent-treatment model). The aims of this study were to explore the abilities of the intermittent therapy model and the continuous treatment model in achieving HBsAg loss in CHB patients receiving PEG-IFN-based therapy and to investigate factors related to HBsAg loss.

## 2 Methods

### 2.1 Study population

**Figure 1:**
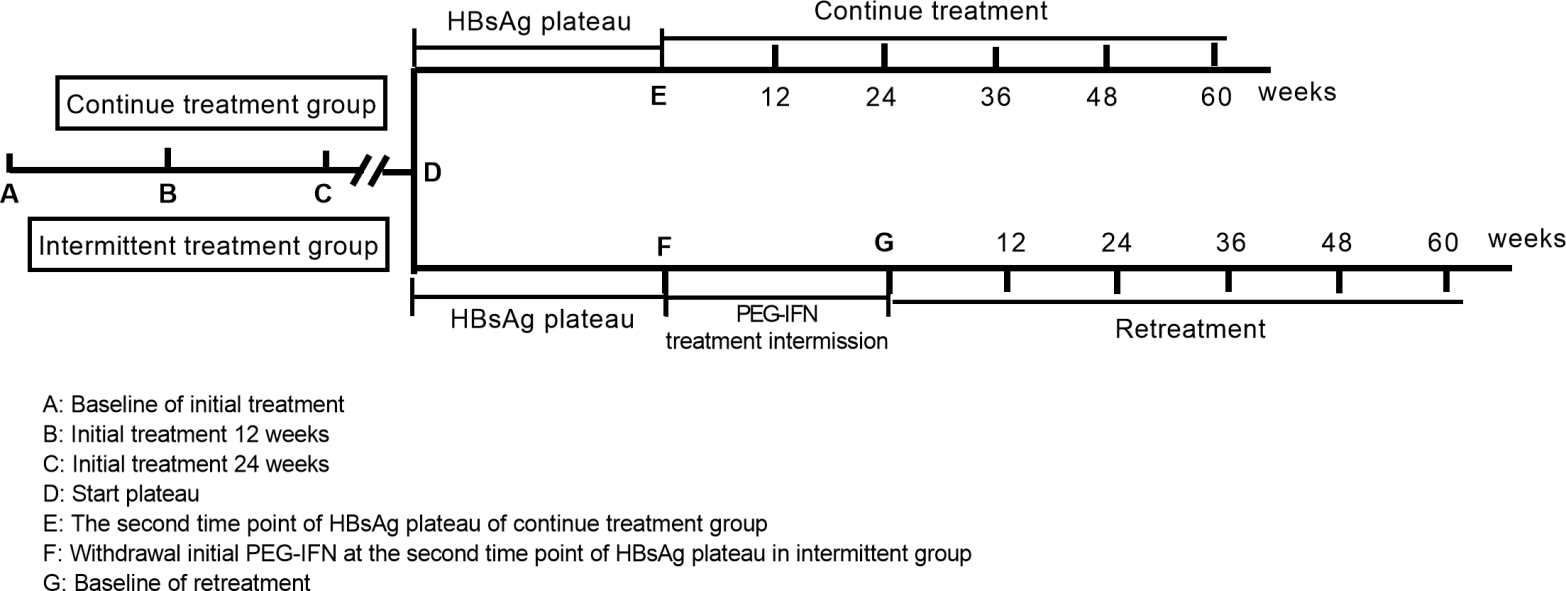
Patient treatment and observation route. After HBsAg decline reached a plateau, patients in the continuous-treatment group received continuous PEG-IFN treatment, whereas patients in the intermittent-treatment group discontinued PEG-IFN for an interval of 3–6 months before restarting PEG-IFN re-treatment.

Patients were enrolled from an observational cohort of CHB patients receiving PEG-IFN-α-based therapy at Beijing Ditan Hospital from 2010 to 2021. Patients reached a plateau in qHBsAg decline after 24 weeks of PEG-IFN-α treatment (180 μg/week administered subcutaneously). Patients in the continuous-treatment group received continuous PEG-IFN therapy, while patients in the intermittent-treatment group discontinued initial PEG-IFN therapy for 3–6 months before resuming treatment. During the observation period, HBV virological and serological parameters and clinical biochemical indicators were monitored every three months, and liver ultrasonography was carried out every 3–6 months (**Figure 1**).

Inclusion criteria: 1) continuous HBsAg positivity for more than six months; 2) qHBsAg decline reaching a plateau after 24 weeks of PEG-IFN-α treatment; 3) achievement of undetectable HBV DNA (HBV DNA load 20 IU/mL) at the plateau phase of qHBsAg decline; 4) after reaching the qHBsAg decline plateau, patients in the continuous-treatment group maintained PEG-IFN therapy for more than 24 weeks, whereas patients in the intermittent-treatment group resumed PEG-IFN retreatment for more than 24 weeks after the treatment interval; and 5) presence of high--risk features, such as a family history of HCC and/or liver cirrhosis.

Exclusion criteria: 1) decompensated liver disease; 2) liver cirrhosis, advanced fibrosis, or hepatocellular carcinoma; 3) contraindications to interferon treatment; 4) HCV, HIV, HAV, or HEV infection; 5) autoimmune liver disease, drug-induced hepatitis, or other liver diseases; and 6) use of immunosuppressive or immunoregulatory drugs.

The study was approved by the Ethics Committee of Beijing Ditan Hospital (Jing Di Lun Ke Zi 2018 No. 023-01).

### 2.2 Outcome measures

The primary measure was the rate of HBsAg loss during 60 weeks of continuous treatment in the continuous-treatment group and during 60 weeks of retreatment in the intermittent-treatment group. Secondary measures were HBsAg seroconversion and the rate of hepatitis B e antigen (HBeAg) loss.

### 2.3 Biochemical indeces

Biochemical indeces were measured using an automatic biochemical analyzer, and peripheral blood counts were tested using an automatic blood cell counting instrument. HBsAg, anti-HBs, HBeAg, and anti-HBe were detected using Abbott Architect i2000 kits (Abbott Laboratories, Abbott Park, IL, USA). HBsAg levels were detected using the Abbott Architect HBsAg QT assay. HBsAg levels below 0.05 IU/mL were defined as HBsAg loss, and anti-HBs ≥ 10 mIU/L was defined as positive. HBV DNA load was tested using the CobasTaqMan 96 real-time quantitative PCR detection reagent (a detection limit of 20 IU/mL) (Roche, Pleasanton, CA, USA). 

### 2.4 Definitions

An HBsAg decline plateau was defined as a decrease in HBsAg level of less than 0.5 log IU/mL compared with the previous time point. 

Patients received IFN therapy when starting antiviral therapy. After at least 24 weeks of treatment, when HBsAg dropped to the plateau, patients voluntarily stopped IFN therapy and used nucleotide analogues (NAs) to maintain inhibition of virus replication. After stopping IFN therapy for 3–6 months, IFN therapy was added again, which was defined as intermittent treatment.

Patients received IFN therapy when starting antiviral therapy. After at least 24 weeks of treatment, when HBsAg dropped to the plateau, patients voluntarily chose to continue IFN therapy, which was defined as intermittent treatment.

### 2.5 Statistical analysis

Categorical variables were expressed as percentages and analyzed using chi-square and non-parametric tests. Continuous variables were expressed as mean ± SD or as median and interquartile ranges (median, Q1-Q3). Differences between the two groups were analyzed using Student’s t-test, analysis of variance, or the Kruskal-Wallis test. To eliminate the effect of deviation in HBsAg levels at the second time point on HBsAg loss during continuous PEG-IFN treatment in the continuous-treatment group and during retreatment in the intermittent-treatment group, propensity score matching (PSM) was performed between the two groups based on HBsAg level at the second time point.

In this study, the exact time of HBsAg loss during continuous PEG-IFN treatment in the continuous-treatment group and during PEG-IFN retreatment in the intermittent-treatment group was determined. Cox regression analysis was used to investigate factors related to HBsAg loss in total patients and in the PSM matched group. Statistical analyses and PSM were performed using SPSS statistics 25.0.

## 3 Results

### 3.1 Study population and baseline characteristics

**Figure 2:**
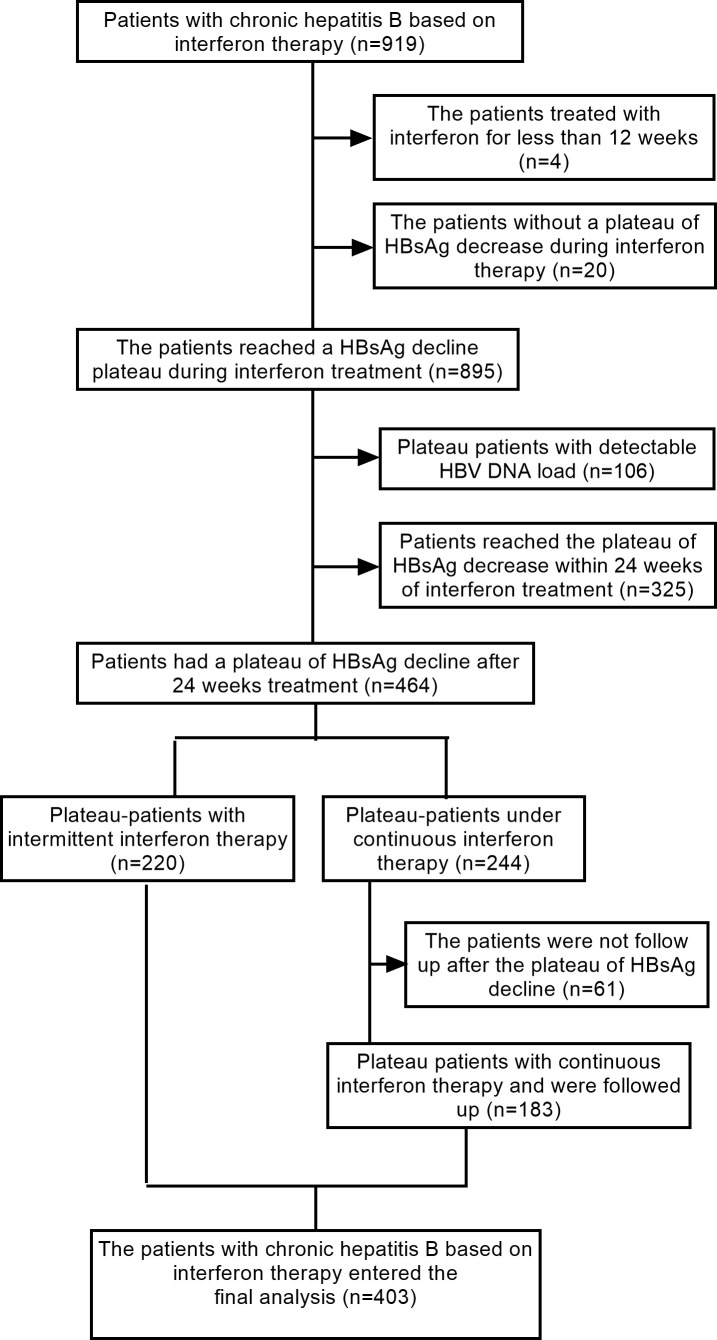
Patient enrollment flowchart.

Among 919 CHB patients receiving PEG-IFN-α therapy, 20 patients with sustained HBsAg decrease during IFN treatment and 4 patients treated for less than 12 weeks were excluded. Among the remaining 895 patients with an HBsAg decline plateau, 106 patients with positive HBV DNA at the HBsAg decline plateau phase and 325 patients whose HBsAg decline plateau occurred within 24 weeks of initial IFN treatment were excluded. This resulted in 464 patients with an HBsAg decline plateau after 24 weeks of IFN treatment, including 220 patients in the intermittent-treatment group and 244 patients in the continuous-treatment group. An additional 61 patients in the continuous-treatment group were excluded because of missing follow-up data after the plateau phase. Finally, 403 patients were included, comprising 220 patients in the intermittent-treatment group and 183 patients in the continuous-treatment group (**Figure 2**).

Among these patients, 288 were male and 115 were female; 97 patients were HBeAg-negative and 306 were HBeAg-positive. A total of 80 patients received PEG-IFN monotherapy, whereas 323 patients received PEG-IFN plus NA therapy (either initial combination therapy or NA treatment added during PEG-IFN therapy), including adefovir (ADV, 19), LAM (61), ETV (230), TDF (3), and two types of NAs (10). Among patients receiving oral antiviral therapy, all received maintenance treatment. Oral antiviral regimens included ADV 10mg/day, LAM 100mg/day, ETV 0.5mg/day, and TDF 300mg/day. In the continuous-treatment group, the numbers of patients receiving ADV, LAM, ETV, TDF, and two types of NAs were 11, 33, 111, 1, and 6, respectively. In the intermittent-treatment group, the numbers of patients receiving ADV, LAM, ETV, TDF, and two types of NAs were 8, 28, 119, 2, and 4, respectively. The baseline indicators, including sex, age, percentage of patients with a family history of HCC, percentage of patients with a family history of liver cirrhosis, percentage of HBeAg-positive patients, and cases with positive HBV DNA, did not differ between the two groups (**Table 1**). Baseline HBsAg levels, HBV DNA load, and liver function parameters before initial PEG-IFN treatment were similar between the two groups (**Table 1**). Although the duration of initial PEG-IFN therapy before reaching the HBsAg decline plateau was not significantly different between the two groups (72.43 weeks vs 74.46 weeks, t = 2.101, p = 0.052), the HBsAg level at the second HBsAg decline plateau time point in the continuous-treatment group was significantly higher than in the intermittent-treatment group (2.7±1.15 vs. 1.80±1.38, t = 7.321, p < 0.001).

**Table 1:** Baseline characteristics of the overall cohort and the PSM-matched population.

Index	Population before PSM					Population matched by PSM				
	All patients (n = 403)	Continuous-treatment group (n = 183)	Intermittent-treatment group (n = 220)	χ^2^ or t/p value	SMD	PSM-matched population (n = 278)	Continuous-treatment group (n = 139)	Intermittent-treatmentgroup (n = 139)	χ^2^ or t/p value	SMD
Age(yrs) (mean±SD)	38.29±8.82	38.60±9.11	38.05±8.59	0.625/0.532	0.06	38.44±8.76	38.45±8.95	38.44±8.61	0.006/0.995	0.001
Male (n, %)	288(71.46%)	126(68.85%)	162(73.64%)	1.121/0.290	0.09	200(71.94%)	99(71.22%)	101(72.66%)	0.071/0.789	0.01
Patients with family history of HCC (n, %)	26(6.45%)	12(6.56%)	14(6.36%)	0.006/0.937	0.008	18(6.47%)	8(5.76%)	10(7.19%)	0.238/0.626	0.059
Patients with family history of liver cirrhosis (n, %)	149(36.97%)	69(37.70%)	80(36.36%)	0.077/0.781	0.028	103(37.05%)	50(35.97%)	53(38.13%)	0.139/0.709	0.045
HBeAg- positive patients (n, %)	306(75.93%)	143(78.14%)	163(74.09%)	0.897/0.344	0.09	215(77.34%)	105(75.54%)	110(79.14%)	0.513/0.474	0.08
Baseline HBV DNA positive (n, %)	244(60.55%)	111(60.66%)	133(60.45%)	0.024/0.877	0.004	162(58.27%)	79(56.83%)	83(59.71%)	0.277/0.599	0.06
Baseline HBV DNA load (log IU/mL) (mean±SD)	5.64±1.80	5.49±1.78	5.76±1.81	-0.713/0.476	0.15	5.65±1.78	5.27±1.74	6.01±1.76	-2.687/0.008	0.42
Baseline HBsAg level of initial PEG-IFN treatment (log IU/mL) (mean±SD)	3.33±0.85	3.35±0.80	3.32±0.90	0.355/0.723	0.03	3.36±0.81	3.36±0.83	3.37±0.82	-0.867/0.340	0.01
ALT level (U/L) [Median(IQR)]	63.60 (30.85, 160.15)	65.10 (30.25,140.93)	60.65 (30.95,184.68)	-1.560/0.120	0.03	55.55 (30.38,155.90)	53.40 (26.10,137.30)	60.60 (31.40,171.80)	-1.401/0.163	0.06
AST level (U/L) [Median(IQR)]	42.20 (25.33, 87.90)	42.15(26.50, 81.25)	42.20 (24.78,90.83)	-1.636/0.103	0.04	41.15 (25.08,86.80)	36.90 (23.70,79.30)	45.00 (25.30,90.40)	-1.547/0.124	0.09
Tbil level (μmol/L) [Median(IQR)]	13.80 (10.60, 18.40)	13.45 (10.75, 18.20)	14.05 (10.55,18.68)	0.264/0.792	0.01	13.80 (11.00,18.13)	13.50 (11.00,18.40)	14.50 (11.00,17.90)	-0.342/0.733	0.05
ALB content (g/L) [Median(IQR)]	45.90 (43.50, 48.58)	45.95 (43.40, 48.90)	45.90 (43.50,48.43)	0.087/0.930	0.02	45.80 (43.50,48.55)	45.90 (43.80,48.90)	45.60 (43.50,48.50)	0.120/0.904	0.03
ALP (U/L) [Median(IQR)]	75.00 (62.20, 90.40)	73.20 (62.20, 83.60)	76.75 (62.28,93.40)	-0.692/0.490	0.07	72.50 (61.98,88.05)	71.60 (62.83,82.75)	73.70 (60.83,90.40)	-0.338/0.736	0.04
TBA (μmol/L) [Median(IQR)]	5.60 (3.00, 10.90)	5.70 (2.70, 11.00)	5.60 (3.10,10.75)	-0.285/0.776	0.03	5.30 (2.90,9.88)	5.70 (2.60,10.70)	5.20 (3.00,9.78)	-0.800/0.425	0.06
GGT (U/L) [Median(IQR)]	38.10 (20.20, 72.50)	38.10 (20.00, 72.00)	38.10 (20.33,79.58)	-0.250/0.802	0.046	36.05 (19.78,67.10)	35.45 (19.78,67.10)	37.95 (19.60,75.35)	-0.419/0.676	0.014
Duration of initial PEG-IFN treatment (weeks) [Median(IQR)]	73.86 (56.86,102.57)	74.46 (55.10, 113.00)	72.43 (58.86, 97.00)	2.101/0.052	0.08	72.57 (57.32, 97.00)	73.57 (59.86,99.14)	71.00 (54.57, 95.00)	1.102/0.727	0.05
HBsAg level at plateau onset (log IU/mL) (mean±SD)	2.05±1.26	2.18±1.20	1.95±1.32	1.825/0.069	0.18	3.34±1.74	2.31±1.18	2.58±1.10	-3.574/0.000	0.24
HBsAg level at the second plateau time point (log IU/mL) (mean±SD)	2.22±1.36	2.73±1.15	1.80±1.38	7.321/0.000	0.72	2.50±1.15	2.51±1.16	2.49±1.14	0.146/0.884	0.02
Early HBsAg response (n, %)	211(52.36%)	113(61.75%)	98(44.55%)	11.853/0.001	0.35	127(45.68%)	81(58.27%)	46(33.09%)	17.758/0.000	0.52
HBsAg loss (n, %)	53(13.15%)	13(7.10%)	40(18.18)	10.734/0.001	0.30	42(15.11%)	12(8.63%)	30(21.58%)	9.087/0.002	0.38
HBsAg seroconversion (n, %)	39(9.68%)	11(6.01%)	28(12.73%)	1.078/0.299	0.21	31(11.15%)	12(8.63%)	19(13.67%)	1.604/0.205	0.13
HBeAg clearance (n, %)	166/306(54.24%)	64/143(44.75%)	102/163(62.57%)	9.748/0.002	0.36	113(40.65%)	53(38.13%)	60(43.17%)	0.731/0.393	0.10

HCC, hepatocellular carcinoma; ALT, Alanine aminotransferase; AST, aspartate aminotransferase; Tbil, total bilirubin; ALB, albumin; ALP, alkaline phosphatase; TBA, total bile acids; GGT, glutamyl transferase; HBsAg, hepatitis B surface antigen; PEG-IFN, pegylated interferon; PSM, propensity score matching; SMD, standardized mean difference.

There were 139 PSM-matched patients in each group. In the matched population, the baseline HBV DNA load and HBsAg level at plateau onset were lower in the continuous-treatment group than in the intermittent-treatment group. HBsAg levels at the post-plateau time point were similar between the two groups (**Table 1**).

### 3.2 Change of HBsAg and HBeAg loss between the two groups

The levels of baseline HBsAg were similar between the two groups (3.32± 0.90 vs. 3.35±0.80, t = 0.355, p = 0.723). Compared with baseline values, HBsAg levels decreased by 1.12 (0.38, 2.01) log IU/mL at the beginning of the plateau phase in the intermittent-treatment group and by 0.99 (0.37, 1.84) log IU/mL in the continuous-treatment group (t=1.676, p = 0.095).

At plateau onset, HBsAg levels were not significantly different between the two groups (1.95±1.32 vs. 2.18±1.20, t=1.825, p = 0.069). However, the percentage of patients with an early HBsAg response, defined as a decrease in HBsAg level > 0.5 log IU/mL within 24 weeks of initial PEG-IFN-α treatment compared with the baseline, was significantly higher in the intermittent-treatment group than in the continuous-treatment group (61.75% vs. 44.55%, χ2 = 11.853, p = 0.001). The HBsAg level at the post-plateau time point was markedly higher in the continuous-treatment group than in the intermittent-treatment group (2.73±1.16 vs. 1.81±1.38, t=7.321, p < 0.001) (**Table 1**).

**Figure 3:**
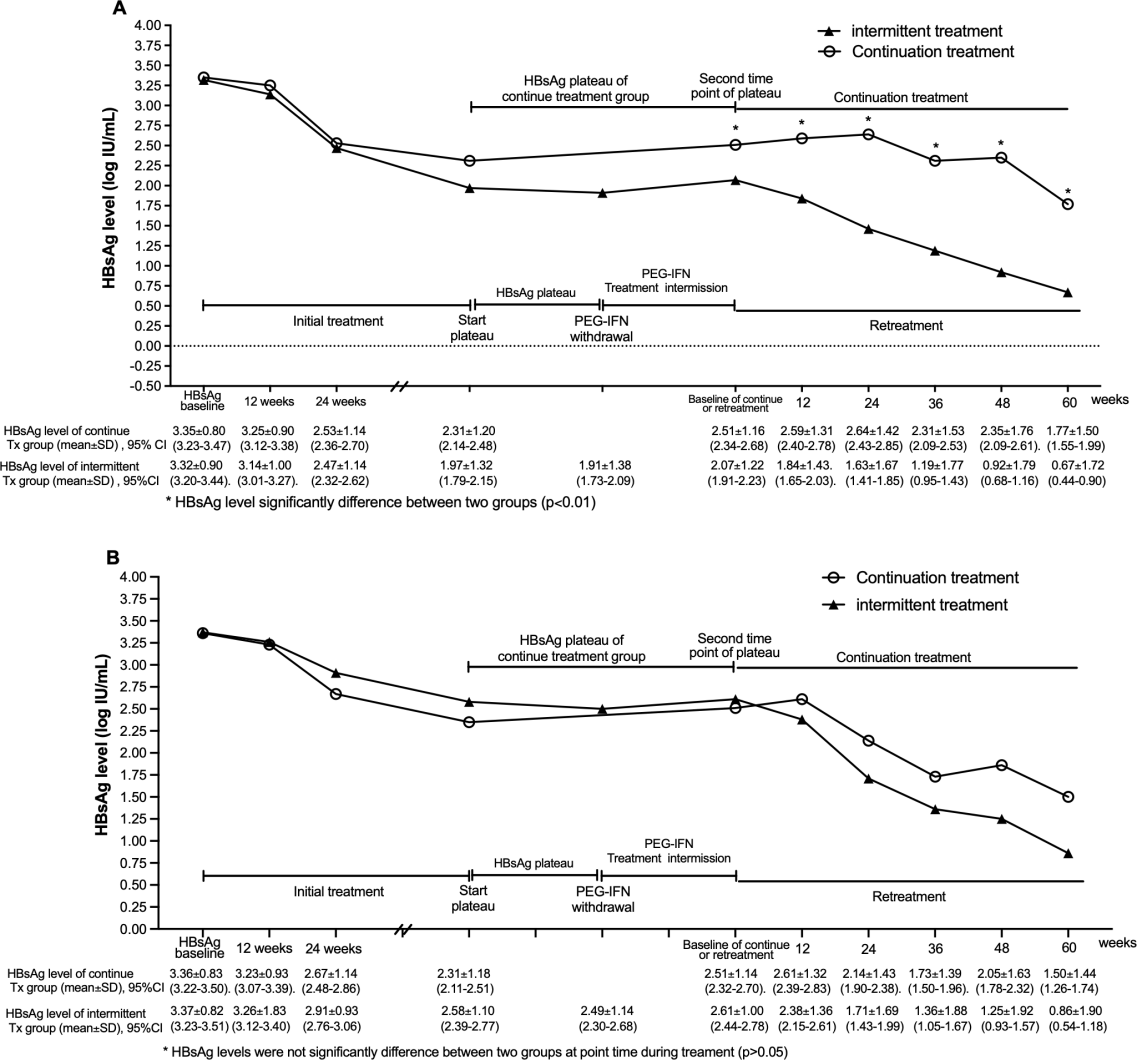
Changes in HBsAg levels during PEG-IFN treatment. **(A)** Overall cohort: HBsAg levels at each time point from plateau onset to the end of treatment were higher in the continuous-treatment group than in the intermittent-treatment group, with significant differences observed during the continuous-treatment phase in the continuous-treatment group and in the retreatment phase in the intermittent-treatment group. **(B)** PSM-matched patients: HBsAg levels at each time point during continuous treatment were higher than during re-treatment in the intermittent-treatment group, although the differences were not significant. Note: “HBsAg baseline”=initial treatment baseline, “plate start”=starting point of the 24 week qHBsAg decline plateau period, “second plate time point”=second time point of the plateau period (3 months from the starting point), “retreatment baseline”=baseline for restarting PEG-IFN treatment after 3–6 months of interruption in the interval group.

In all patients, HBsAg levels at each time point during the retreatment period in the intermittent-treatment group were markedly lower than those during the continuous-treatment period in the continuous-treatment group (p 0.01) (**Figure 3**). In the matched patients, although HBsAg levels were similar at the time point after the post-plateau between the two groups, HBsAg levels during retreatment in the intermittent-treatment group was lower than those during the continuous-treatment period in the continuous-treatment group (**Figure 3**).

**Figure 4:**
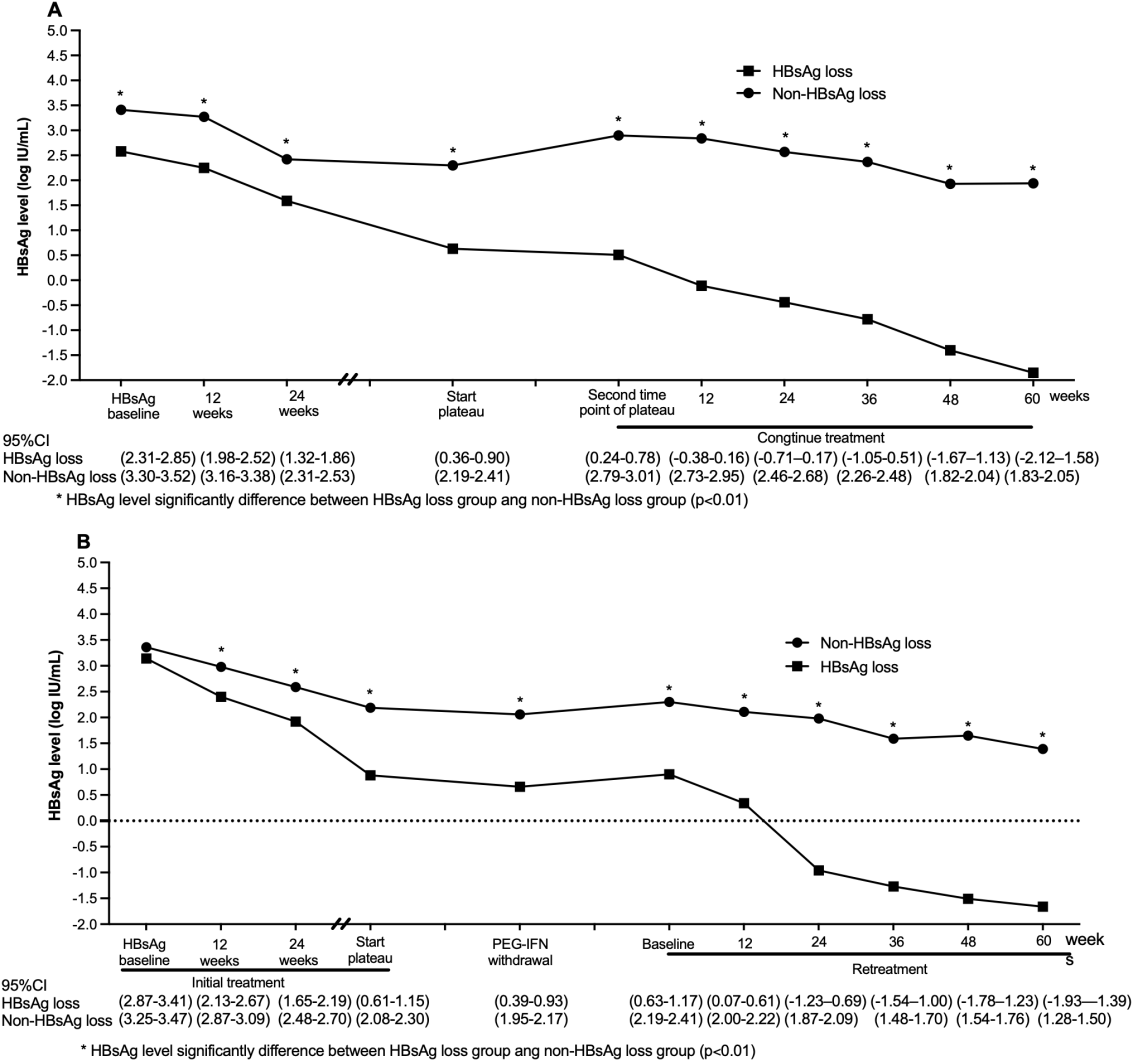
Changes in HBsAg levels in patients with HBsAg loss and non-HBsAg loss during continuous treatment and retreatment **(A)**: Continuous-treatment group; **(B)**: Intermittent-treatment group. HBsAg levels at each time point throughout the treatment in patients achieving HBsAg loss were significantly lower than in patients without HBsAg loss. Note: “plate phase”=plateau period (24 weeks), “post plateau”=time node for continuing/restarting PEG-IFN treatment after the plateau period.

Among the total population, patients who achieved HBsAg loss had significantly lower HBsAg levels at all treatment time points than patients who did not achieve HBsAg loss (**Figure 4**). The rate of HBeAg loss in patients with continuous treatment was markedly lower than that in patients with intermittent treatment [44.8% (64/143) vs. 62.6% (102/163), χ2 = 9.748, p = 0.002] (**Table 1**).

### 3.3 HBsAg loss and HBsAg seroconversion

Among all 403 patients, 53 (13.15%) achieved loss of HBsAg, and 39 (9.68%) seroconversion. Although the percentage of HBsAg loss was significantly lower in patients with continuous treatment [7.10% (13/183)] than in patients with intermittent treatment [18.18% (40/220)] (χ2 = 10.734, p = 0.001), there was no statistical difference in the HBsAg seroconversion rate [12.73% (28/220) vs. 6.01% (11/183), χ2 = 1.078, p = 0.299] (**Table 1**). Among the 211 patients who achieved an early HBsAg response, the rate of HBsAg loss was 16.11%. In patients with an early HBsAg response, the rate of HBsAg loss was significantly higher in the intermittent-treatment group than in the continuous-treatment group [7.1% (8/113) vs. 26.5% (26/98), χ2 = 14.690, p < 0.001] (**Table 1**).

**Figure 5:**
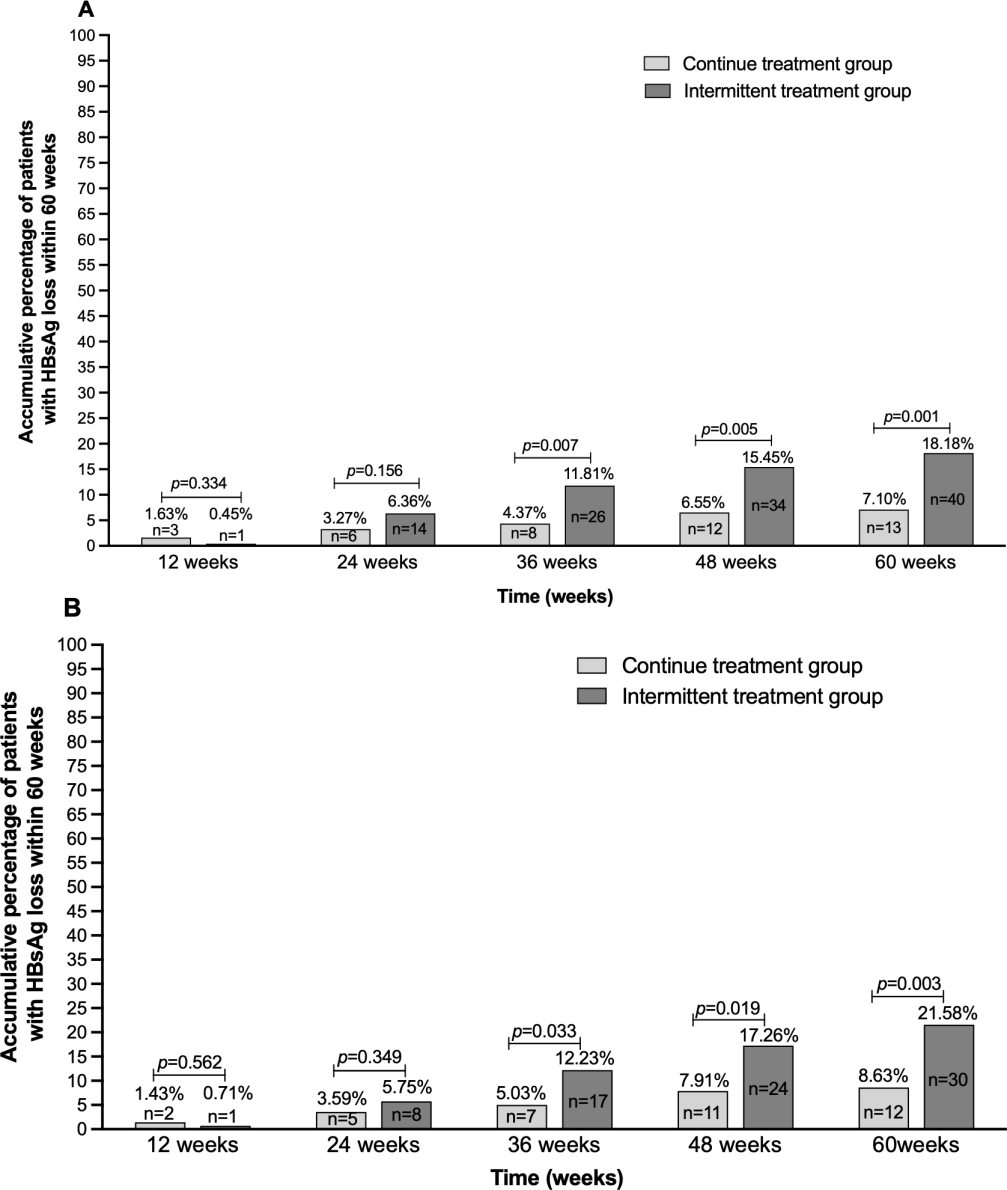
Cumulative incidence of HBsAg loss in the continuous-treatment group and intermittent-treatment group. In both the overall cohort **(A)** and the PSM-matched population **(B)**, cumulative HBsAg loss rates from 36 weeks of retreatment were higher in the intermittent-treatment group than in the continuous-treatment phase in the continuous-treatment group. Note: In the intermittent treatment group, “12/24/36/48/60 weeks”=follow-up time points for restarting PEG-IFN treatment after the plateau period; In the continuous treatment group, “12/24/36/48/60 weeks”=the follow-up time point for continuing PEG-IFN treatment after the plateau period.

In the PSM-matched population, the rate of HBsAg loss was 15.11% (42/278), with the intermittent-treatment group achieving a significantly higher rate than the continuous-treatment group [21.58% (30/139) vs. 8.63% (12/139), respectively χ2 = 10.734, p = 0.002] (**Table 1**). In both the overall patient cohort and the matched population, the cumulative rates of HBsAg loss at weeks 36, 48, and 60 during the retreatment period were significantly higher in the intermittent-treatment group than in the continuous-treatment group (p0.01, **Figure 5**).

### 3.4 Factors related to HBsAg loss

In the total population, univariate Cox regression analysis revealed that patients with lower glutamyl transferase (GGT) levels, lower baseline HBsAg levels, and lower HBsAg levels during the plateau phase (including plateau onset and post-plateau time points), and patients receiving the intermittent-treatment model were markedly correlated to HBsAg loss during treatment after the plateau phase. The results of multivariate COX regression analysis showed that the low level of HBsAg at the post-plateau time point (OR = 0.173, 95% CI 0.068-0.442, p < 0.001) and intermittent treatment (OR = 0.309, 95% CI 0.118-0.808, p = 0.017) were independent factors for predicting the occurrence of HBsAg loss (**Table 2**).

**Table 2:** Clinical characteristics and factor analysis of HBsAg loss and non-HBsAg loss in the overall cohort.

Index	Patient groups			Cox regression			Multivariate Cox regression		
	HBsAg loss (n = 53)	Non-HBsAg loss (n = 350)	χ^2^ or t/p value	OR	95% CI	p value	OR	95% CI	p value
Age(yrs) (mean±SD)	37.32±8.86	38.44±8.82	-0.863/0.389	0.986	0.955-1.017	0.370			
Male (n, %)	42(79.24%)	246(70.28%)	1.812/0.178	0.722	0.371-1.403	0.337			
Patients with family history of HCC (n, %)	5(9.43%)	21(6.00%)	0.899/0.364	0.989	0.958-1.020	0.391			
Patients with family history of liver cirrhosis (n, %)	14(26.42%)	135(38.57%)	2.919/0.088	0.698	0.364-1.136	0.268			
HBeAg-positive patients (n, %)	35(66.03%)	271(77.42%)	3.268/0.071	0.635	0.359-1.122	0.118			
Baseline HBVDNA positive (n, %)	30(56.60%)	214(56.31%)	0.702/0.402	0.907	0.510-1.612	0.739			
Baseline HBV DNA load (log IU/mL) (mean±SD)	5.30±1.92	5.68±1.78	-1.239/0.216	0.965	0.879-1.058	0.445			
Baseline HBsAg level of initial PEG-IFN treatment (log IU/mL) (mean±SD)	3.00±0.97	3.38±0.82	-3.048/0.002	0.695	0.522-0.926	0.013			
Time of initial PEG-IFN treatment (weeks) [Median(IQR)]	99.14(66.57, 117.93)	71.78(54.82, 96.46)	2.538/0.012	1.008	0.999-1.017	0.099			
Early response to HBsAg (n, %)	34(64.15%)	177(50.57%)	3.403/0.065	1.648	0.940-2.890	0.081			
Intermittent interferon therapy (n, %)	40(75.47%)	180(51.42%)	10.734/0.001	1.895	1.013-3.545	0.046	3.236	1.238-8.475	0.017
ALT level(U/L) [Median(IQR)]	38.00(24.00,122.20)	69.20(31.80,162.10)	-0.083/0.934	1.000	0.999-1.001	0.930			
AST level(U/L) [Median(IQR)]	29.00(22.00,65.55)	45.00(26.80,89.30)	0.459/0.647	1.001	0.999-1.002	0.580			
Tbil level(μmol/L) [Median(IQR)]	13.90(11.75,19.25)	13.80(10.30,18.40)	-0.206/0.837	1.003	0.965-1.041	0.891			
ALB content(g/L) [Median(IQR)]	46.60(43.75,49.20)	45.90(43.50,48.50)	1.267/0.206	1.050	0.969-1.138	0.234			
ALP (U/L) [Median(IQR)]	67.10(49.80,83.20)	76.45(64.30,91.00)	-1.582/0.115	0.987	0.971-1.003	0.117			
TBA (μmol/L) [Median(IQR)]	4.20(2.50,8.35)	5.75(3.10,11.40)	-3.333/0.001	0.945	0.882-1.012	0.104			
GGT (U/L) [Median(IQR)]	26.50(20.20,43.85)	39.65(20.45,79.63)	-4.877/0.000	0.984	0.971-0.998	0.024			
HBsAg level at plateau onset (log IU/mL) (mean±SD)	0.81±1.10	2.24±1.18	-8.131/0.000	0.473	0.386-0.581	0.000			
HBsAg level at the second plateau time point (log IU/mL) (mean±SD)	0.62±1.14	2.47±1.22	-10.373/0.000	0.371	0.290-0.475	0.000	0.173	0.068-0.442	0.000

HCC, hepatocellular carcinoma; ALT, Alanine aminotransferase; AST, aspartate aminotransferase; Tbil, total bilirubin; ALB, albumin; ALP, alkaline phosphatase; TBA, total bile acids; GGT, glutamyl transferase; HBsAg, hepatitis B surface antigen; PEG-IFN, pegylated interferon.

In the matched population, the Cox regression analysis also showed that baseline HBsAg levels, HBsAg levels during the plateau phase (including plateau onset and post-plateau time points), early response to HBsAg, and intermittent treatment were associated with HBsAg loss. The multivariate Cox regression analysis showed that the low level of baseline HBsAg (OR = 2.027, 95% CI 1.024-4.013, p = 0.043), low HBsAg level at the second plateau time point (OR = 0.051, 95% CI 0.011-0.246, p < 0.001), and intermittent treatment (OR = 5.173, 95% CI 1.714-15.613, p = 0.004) were independent factors predicting the occurrence of HBsAg loss (**Table 3**).

**Table 3:** Clinical characteristics and factor analysis of HBsAg loss and non-HBsAg loss in the PSM-matched population.

Index	Patients groups			Cox regression			Multivariate Cox regression		
	HBsAg loss (n = 42)	Non-HBsAg loss (n = 236)	χ^2^ or t/p value	OR	95% CI	P value	OR	95% CI	P value
Age (yrs) (mean±SD)	36.76±9.02	38.74±8.70	0.351/0.178	0.973	0.935-1.013	0.179			
Male (n, %)	33(78.57%)	167(70.76%)	1.077/0.299	0.660	0.300-1.452	0.302			
HBeAg-positive patients (n, %)	31(73.81%)	184(77.96%)	0.351/0.553	0.796	0.375-1.692	0.554			
Baseline HBV DNA positive (n, %)	21(50.00%)	141(59.74%)	1.587/0.208	0.640	0.318-1.287	0.210			
Baseline HBV DNA load (log IU/mL) (mean±SD)	5.52±1.98	5.67±1.76	0.368/0.713	0.953	0.738-1.231	0.711			
Baseline HBsAg level of initial PEG-IFN treatment (log IU/mL)	3.02±1.03	3.40±0.77	2.295/0.026	0.599	0.412-0.869	0.007	0.493	0.249-0.977	0.043
Time of initial PEG-IFN treatment (weeks) [Median(IQR)]	97.21(64.96, 115.50)	70.42(55.10, 92.14)	1.102/0.272	1.008	0.997-1.019	0.147			
Early response to HBsAg (n, %)	26(61.90%)	101(42.79%)	5.246/0.022	2.172	1.107-4.262	0.024			
Intermittent interferon therapy (n, %)	30(71.42%)	109(46.18%)	9.087/0.003	2.913	1.423-5.965	0.003	5.173	1.714-15.613	0.004
ALT level(U/L) Median(IQR)	38.45(31.03,134.20)	61.60(29.90,157.50)	-0.477/0.634	1.000	0.999-1.002	0.635			
AST level(U/L) Median(IQR)	30.10(24.28,68.93)	45.10(25.30,89.15)	-0.439/0.663	1.001	0.999-1.003	0.431			
Tbil level(μmol/L) Median(IQR)	15.20(12.05,20.98)	13.75(10.90,17.75)	-0.792/0.430	1.018	0.973-1.066	0.431			
ALB content(g/L) Median(IQR)	46.70(44.80,49.53)	45.60(43.50,48.25)	-1.499/0.136	1.078	0.977-1.189	0.136			
ALP (U/L) Median(IQR)	67.10(53.40,83.60)	73.40(63.65,88.20)	0.883/0.379	0.992	0.973-1.010	0.377			
TBA (μmol/L) Median(IQR)	4.50(2.50,8.40)	5.30(2.90,10.10)	1.154/0.250	0.955	0.886-1.028	0.222			
GGT (U/L) Median(IQR)	33.00(20.20,44.80)	37.70(18.70,71.45)	1.692/0.093	0.988	0.974-1.002	0.102			
HBsAg level at plateau onset (log IU/mL) (mean±SD)	1.12±1.00	2.54±1.06	7.971/0.000	0.343	0.243-0.484	0.000			
HBsAg level at the second plateau time point (log IU/mL) (mean±SD)	0.94±1.03	2.77±0.91	11.761/0.000	0.184	0.116-0.293	0.000	0.051	0.010-0.246	0.000

ALT, Alanine aminotransferase; AST, aspartate aminotransferase; Tbil, total bilirubin; ALB, albumin; ALP, alkaline phosphatase; TBA, total bile acids; GGT, glutamyl transferase; HBsAg, hepatitis B surface antigen; PEG-IFN, pegylated interferon; PSM, propensity score match.

## 4 Discussion

HBsAg loss, whether spontaneous or achieved through antiviral therapy, is the key indicator of favorable long-term outcomes and predicts the lowest incidence of HCC (Chu and Liaw, 2010). HBsAg loss has become the ideal endpoint of antiviral treatment and treatment goal (European Association for the Study of the Liver, 2017; Terrault et al., 2018; Chinese Society of Hepatology, Chinese Society of Infectious Diseases, 2022). In chronic HBV infection, HBV and its antigens can inhibit host immune cell functions, such as blocking the signaling pathway of IFN-α (Kouwaki et al., 2017), inhibiting the formation and maturation of dendritic cells (DC) (Yonejima et al., 2019), inducing the production of regulatory T cells (Luangsay et al., 2015), and inhibiting the secretion of IFN-γ by NK cells (Wijaya et al., 2019). These effects make it very difficult for patients with CHB to recover from HBV infection. The spontaneous HBsAg loss rate in inactive carriers is only 1%-1.5% per year (Manno et al., 2004; Chu and Liaw, 2007). Even among CHB patients who received first-line oral NA antiviral treatment, the annual rate of HBsAg loss was only 0.22% (Hsu et al., 2021). Unlike NAs, which do not affect viral antigen production, IFN can directly inhibit viral replication and viral antigen synthesis by inducing the expression of antiviral proteins and inducing specific, non-hepatotoxic degradation of nuclear HBV covalently closed circular DNA (cccDNA) (Lucifora et al., 2014). IFN also exerts immunoregulatory effects (Randall and Goodbourn, 2008). Many studies have shown that interferon-based therapy achieves higher clinical cure rates than NA therapy in CHB patients (Li et al., 2017b; 2019; 2024; Choi et al., 2022).

HBsAg loss indicates a decrease in the quantity and transcriptional activity of cccDNA in the liver (Moucari et al., 2009; van Campenhout and Janssen, 2015). During antiviral therapy with interferon, the half-life of infected hepatocyte clearance varies greatly among patients (Sypsa et al., 2005), and prolonged therapy is often needed to improve HBsAg loss rates in patients receiving PEG-IFN treatment (Li et al., 2021), even in dominant patients who have lower baseline HBsAg levels or an early HBsAg response (Hu et al., 2018; Yan et al., 2018). To obtain HBsAg loss, current guidelines therefore recommend prolonged PEG-IFN treatment in patients with favorable responses (Chinese Society of Hepatology, Chinese Society of Infectious Diseases, 2022).

However, the majority of patients fail to achieve HBsAg loss because HBsAg levels do not continue to decline throughout IFN treatment. In CHB patients switched from NA treatment to PEG-IFN therapy, the rate of HBsAg loss after 94 weeks of PEG-IFN treatment was 20.7%, which was not markedly higher than the rate after 48 weeks (14.4%) (Hu et al., 2018). Even among patients with favorable baseline characteristics, including baseline HBsAg <1500 IU/mL and HBsAg <200 IU/mL at 24 weeks of treatment, extending treatment from 48 weeks to 96 weeks increased the HBsAg loss rate only from 36.95% to 58.69% (Hu et al., 2018). Our previous studies have shown that, during IFN treatment in CHB patients, HBsAg levels often decline to a plateau, after which further decreases in HBsAg becomes difficult to achieve. Once the decline in HBsAg reaches a plateau, it is difficult for the majority of patients to achieve a HBsAg decrease through continuous treatment (Li et al., 2021). Under these circumstances, a clinical decision must be made regarding whether to continue IFN treatment or to suspend therapy and restart IFN treatment after an interval period. This study compared prolonged treatment with intermittent treatment to determine which strategy was more favorable for achieving HBsAg loss.

A previous study showed that IFN receptor expression was significantly reduced after three months of IFN-α treatment in responders with chronic hepatitis C, whereas receptor expression was not affected in non-responders (Massirer et al., 2004). In the treatment of CHB, the efficacy of PEG-IFN-α may be limited by its depleting effect on CD8+ T cells (Micco et al., 2013). HLADR+CD38+ T lymphocytes play an effective role in immune activation and viral clearance (Nienhold et al., 2020; Wang et al., 2020). There are two heterogeneous subsets of HLA-DR+CD38+: HLADR+CD38dim and HLA-DR+CD38hi. The former expresses strong cytotoxic potential and is less sensitive to apoptosis, while the latter is in a state of overactivation or depleted immune disorder, and its cytotoxic function is impaired (Du et al., 2021). We previously found that in the process of long-term treatment of PEG-IFN-α in CHB patients, the proportion of HLA-DR+CD38dim subsets significantly increased at first and then gradually decreased, while the percentage of HLA-DR+CD38hi subsets increased markedly. Serum HBsAg levels were negatively correlated with the percentage of HLA-DR+CD38hi subset, and persistently high proportions of HLA-DR+CD38hi subsets were related to the occurrence of an HBsAg decline plateau (Lin et al., 2023). These findings suggest that the therapeutic effects of IFN therapy may not be sustained and the decline of HBsAg reaches a plateau.

In this study, all patients experienced an HBsAg decline plateau after 24 weeks of IFN treatment. Once HBsAg decline reached a plateau, patients either chose to continue PEG-IFN treatment or temporarily stop IFN therapy for 3–6 months before retreatment. Before initial IFN therapy, there were no statistically significant differences between the two groups at baseline. Although a higher percentage of patients in the continuous-treatment group achieved an early HBsAg response during initial IFN therapy than those in the intermittent-treatment group, this may have influenced their decision to continue therapy.

In the overall population, the HBsAg loss rate after plateau onset was only 7.10% in the continuous-treatment group, which was significantly lower than that in patients receiving intermittent treatment (18.18%). In the intermittent-treatment group, the suspension of IFN therapy may have allowed recovery of immune cell function (Lin et al., 2023). In this study, in the intermittent treatment group, HBsAg levels at the time of discontinuation of initial interferon treatment were lower than in the continuous-treatment group at the second plateau time point before continuous treatment, which may have influenced the decision to choose retreatment after the interval. In order to reduce the impact of differences in HBsAg levels at the post-plateau time point between the two groups on subsequent treatment outcomes, PSM matching between the two groups was performed based on HBsAg levels at the abovementioned time points. Among the 278 matched patients, the rate of HBsAg loss in the intermittent-treatment group was significantly higher than in the continuous-treatment group (21.58% vs. 8.63%, respectively p = 0.002). These results indicate that compared with continuous therapy after a HBsAg decline plateau, intermittent treatment can significantly improve the rate of HBsAg loss. In the overall population, the intermittent-treatment group also had significantly higher percentage of patients achieving HBeAg loss than the continuous-treatment group.

The Cox model not only takes into account the outcome of the event, but also incorporates information provided by the survival time. In order to explore whether IFN therapy contributes to the occurrence of HBsAg loss, patients were divided into an HBsAg loss group and a non-HBsAg loss group. Compared with the non-HBsAg loss group, patients in the HBsAg loss group had significantly lower baseline HBsAg levels, lower HBsAg levels at plateau time points, and lower GGT levels. The HBsAg loss group had more patients receiving intermittent treatment, and had longer IFN treatment time before continuous treatment or initial IFN treatment. Multivariate Cox regression analysis showed that the low HBsAg level at the post-plateau time point and intermittent treatment were independent factors associated with HBsAg loss. In the PSM-matched population, multivariate Cox regression analysis found that the low level of baseline HBsAg, low HBsAg level at the second plateau time point, and intermittent treatment were independent factors predicting the occurrence of HBsAg loss.

In IFN treatment for CHB, favorable conditions, such as lower baseline HBsAg levels or an early HBsAg response to the treatment, together with prolonged IFN treatment, are important predictors of HBsAg loss (Hu et al., 2018; Yan et al., 2018; Li et al., 2021). However, not all patients with favorable characteristics achieve HBsAg loss, and continuous treatment after an HBsAg decline plateau appears to have limited the effect in improving HBsAg loss rates (Hu et al., 2018; Li et al., 2021). Our results suggest that in patients with low baseline HBsAg levels, especially those who achieve a favorable HBsAg response during initial IFN treatment, once HBsAg decline reaches a plateau, it is highly recommended that IFN treatment be suspended for an interval of 3–6 month before IFN retreatment.

This study has several limitations. First, it was a retrospective cohort study, and patients were not randomized to treatment groups. Due to the non-randomized design, preference-based treatment allocation is inherently susceptible to selection bias, as unmeasured differences may have existed between the two groups. Moreover, despite propensity score matching, residual confounding from unmeasured factors, such as immune status and NA adherence, may still have influenced HBsAg loss rates. Excluding patients with HBV DNA rebound during off-treatment intervals may have enriched the intermittent group with better immune control, potentially overestimating the benefit of intermittent therapy. Therefore, well-designed randomized controlled trials with comprehensive covariate collection are needed to confirm our findings. However, all baseline characteristics were similar between the two groups, and PSM was used to eliminate the effect of deviation in HBsAg levels at the second HBsAg decline plateau time point. Although the baseline HBV DNA load was significantly different between the two groups, all enrolled patients achieved undetectable HBV DNA at the HBsAg decline plateau, thus reducing the effect of baseline HBV DNA on post-plateau treatment outcomes. During the study period, patients in the intermittent group who experienced viral rebound of HBV DNA (>20 IU/mL) during the off-treatment intervals were excluded. The exclusion of these re-positive patients, who likely had poorer immunological control, may have resulted in the enrichment of the intermittent group with patients demonstrating better immune control. There was no data on HBV genotypes because these tests were not routinely performed before treatment. The effects of family history and medication history on changes of HBsAg during treatment were not analyzed. Future prospective studies should include immune indicator testing to validate the mechanisms of immune exhaustion and recovery, providing more direct evidence support for the application of interval therapy. In this study, patients with liver cirrhosis were not included. Exclusion of these patients may lead to population heterogeneity and potentially affect the results. In future studies, we will further explore the benefits of different treatment strategies in this specific population. Our study did not systematically collect baseline liver stiffness measurements or FIB-4 data. Although patients with cirrhosis or clinically suspected advanced fibrosis (F3) were excluded based on ultrasound and routine testing, the exact distribution of fibrosis stages (F0–F2) remains unknown. Thus, we cannot completely rule out residual confounding by baseline fibrosis status. Future prospective studies should incorporate non-invasive fibrosis markers to validate our findings.

In summary, intermittent treatment can significantly improve the efficacy of IFN therapy and enhance the occurrence of HBsAg loss.

## Data Availability

The raw data supporting the conclusions of this article will be made available by the authors, without undue reservation.
